# Treatment with methyl-β-cyclodextrin prevents mechanical allodynia in resiniferatoxin neuropathy in a mouse model

**DOI:** 10.1242/bio.039511

**Published:** 2018-12-21

**Authors:** Chih-Lung Lin, Chin-Hong Chang, Ying-Shuang Chang, Shui-Chin Lu, Yu-Lin Hsieh

**Affiliations:** 1 Department of Neurosurgery, Kaohsiung Medical University Hospital, Kaohsiung 80708, Taiwan; 2 Faculty of Medicine, Graduate Institute of Medicine, College of Medicine, Kaohsiung Medical University, Kaohsiung 80708, Taiwan; 3 Department of Surgery, Chi Mei Medical Center, Tainan 71004, Taiwan; 4 Department of Anatomy, School of Medicine, College of Medicine, Kaohsiung Medical University, Kaohsiung 80708, Taiwan; 5 Department of Medical Research, Ultrastructural Laboratory, Kaohsiung Medical University Hospital, Kaohsiung 80708, Taiwan; 6 Department of Medical Research, Kaohsiung Medical University Hospital, Kaohsiung 80708, Taiwan

**Keywords:** Small-fiber neuropathy, Transient receptor potential vanilloid subtype 1, Prostatic acid phosphatase, Cholesterol, Methyl-β-cyclodextrin, Phosphatidylinositol 4, 5-bisphosphate

## Abstract

Specialized microdomains which have cholesterol-rich membrane regions contain transient receptor potential vanilloid subtype 1 (TRPV1) are involved in pain development. Our previous studies have demonstrated that the depletion of prostatic acid phosphatase (PAP) – a membrane-bound ectonucleotidase ­– and disordered adenosine signaling reduce the antinociceptive effect. The role of membrane integrity in the PAP-mediated antinociceptive effect in small-fiber neuropathy remains unclear, especially with respect to whether TRPV1 and PAP are colocalized in the same microdomain which is responsible for PAP-mediated antinociception. Immunohistochemistry was conducted on the dorsal root ganglion to identify the membrane compositions, and pharmacological interventions were conducted using methyl-β-cyclodextrin (MβC) – a membrane integrity disruptor that works by depleting cholesterol – in pure small-fiber neuropathy with resiniferatoxin (RTX). Immunohistochemical evidence indicated that TRPV1 and PAP were highly colocalized with flotillin 1 (66.7%±9.7%) and flotillin 2 (73.7%±6.0%), which reside in part in the microdomain. MβC mildly depleted PAP, which maintained the ability to hydrolyze phosphatidylinositol 4,5-bisphosphate [PI(4,5)P2] and delayed the development of mechanical allodynia. MβC treatment had no role in thermal transduction and neuronal injury following RTX neuropathy. In summary, this study demonstrated the following: (1) membrane cholesterol depletion preserves PAP-mediated antinociception through PI(4,5)P2 hydrolysis and (2) pain hypersensitivity that develops after TRPV1(+) neuron depletion-mediated neurodegeneration following RTX neuropathy is attributable to the downregulation of PAP analgesic signaling.

## Abstract (precis)

**Summary:** The role and mechanism of cholesterol-rich membrane integrity in pain development for small-fiber neuropathy remains unclear. Depletion of membrane cholesterol contents preserves functional PAP profiles and the antinociceptive effect after RTX neuropathy.

## INTRODUCTION

A specialized microdomain which has cholesterol-rich membrane regions contains several transmembrane molecules that modulate cellular physiology. Evidence suggests that microdomains serve as organizing centers and are correlated to neurodegeneration (Sonnino et al., 2014), peripheral neuropathy (Gambert et al., 2017; Lee et al., 2014), neuronal interactions (Leyton and Hagood, 2014; Marchenkova et al., 2016) and synaptic transmission (De Chiara et al., 2013). Alteration of the membrane composition of a microdomain is associated with the pathogenesis of neurodegeneration (Gambert et al., 2017; Lee et al., 2014; Sonnino et al., 2014). Disrupting the membrane integrity causes distinct effects such as reversing the cytotoxicity of antitumor drugs (Adinolfi et al., 2013), antagonizing hyperalgesia (Dina et al., 2005) and inhibiting endocannabinoid-mediated analgesic systems (Rossi et al., 2012). These effects imply that microdomains may affect neuronal regulation, particularly in neuronal antinociception. Notably, the transmembrane isoform of prostatic acid phosphatase (PAP) was previously documented in microdomains (Quintero et al., 2007); PAP has ectonucleotidase properties (Sowa et al., 2009) that can hydrolyze extracellular adenosine monophosphate (AMP) to the antinociceptive agonist adenosine (Street and Zylka, 2011; Zylka et al., 2008). The antinociceptive effect of PAP that prevents pain hypersensitivity has been thoroughly documented, and our previous research confirmed that PAP neuropathology results in the loss of antinociception; that is, injured PAP(+) neurons mediate pain hypersensitivity (Wu et al., 2016) through disorder of adenosine signaling (Kan et al., 2018). However, the mechanism involved in this effect remains uncertain and requires further investigation. Research is necessary to determine whether a transmembrane molecule exists that colocalizes and interacts with PAP in an integrity of membrane structure to modulate PAP-mediated antinociception. If one does exist, transient receptor potential vanilloid subtype 1 (TRPV1) is a candidate because PAP mediates antinociception by reducing TRPV1 activity (Sowa et al., 2010), and TRPV1-mediated nociception requires TRPV1 on membrane integrity (Marchenkova et al., 2016; Saghy et al., 2015; Szőke et al., 2010).

TRPV1 is a non-selective ion channel and a polymodal nociceptor that responds to thermal nociception (Caterina et al., 2000; Caterina et al., 1997); depletion of TRPV1(+) neurons using the selective neurotoxic agent resiniferatoxin (RTX) leads to thermal hypoalgesia (Hsieh et al., 2012b; Karai et al., 2004). We established a mouse model of pure small-fiber neuropathy that causes mechanical allodynia in addition to reducing intraepidermal nerve fibers (IENFs) and inducing thermal hypoalgesia (Hsieh et al., 2012a;  2008, 2012b; Pan et al., 2003). On the basis of this RTX neuropathy model, we demonstrated that mechanical allodynia and thermal hypoalgesia were induced concurrently through distinct pathways (Hsieh et al., 2012a, b; Lin et al., 2013) and by different neurotrophin-dependent receptors (Hsieh et al., 2018). In addition, PAP was determined to mediate antinociception through hydrolysis of phosphatidylinositol 4,5-bisphosphate [PI(4,5)P2], which was found to reduce TRPV1-mediated nociception (Sowa et al., 2010). By contrast, the depletion of TRPV1(+) neurons has been demonstrated to induce ATP-sensitized P2X3 (Hsieh et al., 2012a), which has been demonstrated to colocalize with PAP (Wu et al., 2016). Collectively, these findings suggest that TRPV1(+) neuron-dependent neuropathic manifestation involves the close interaction of TRPV1 and PAP because of colocalization and that these two molecules are similarly neurophysiologically modulated (i.e. nerve growth factor-mediated tyrosine receptor kinase A signaling) (Wu et al., 2016). In this study, we investigated the following concerns: (1) whether PAP and TRPV1 that are colocalized in the same cholesterol-rich microdomain modulate antinociception and nociceptive transduction and (2) the consequences of disrupting the integrity of a microdomain containing PAP and TRPV1 in RTX neuropathy.

**Fig. 1.**
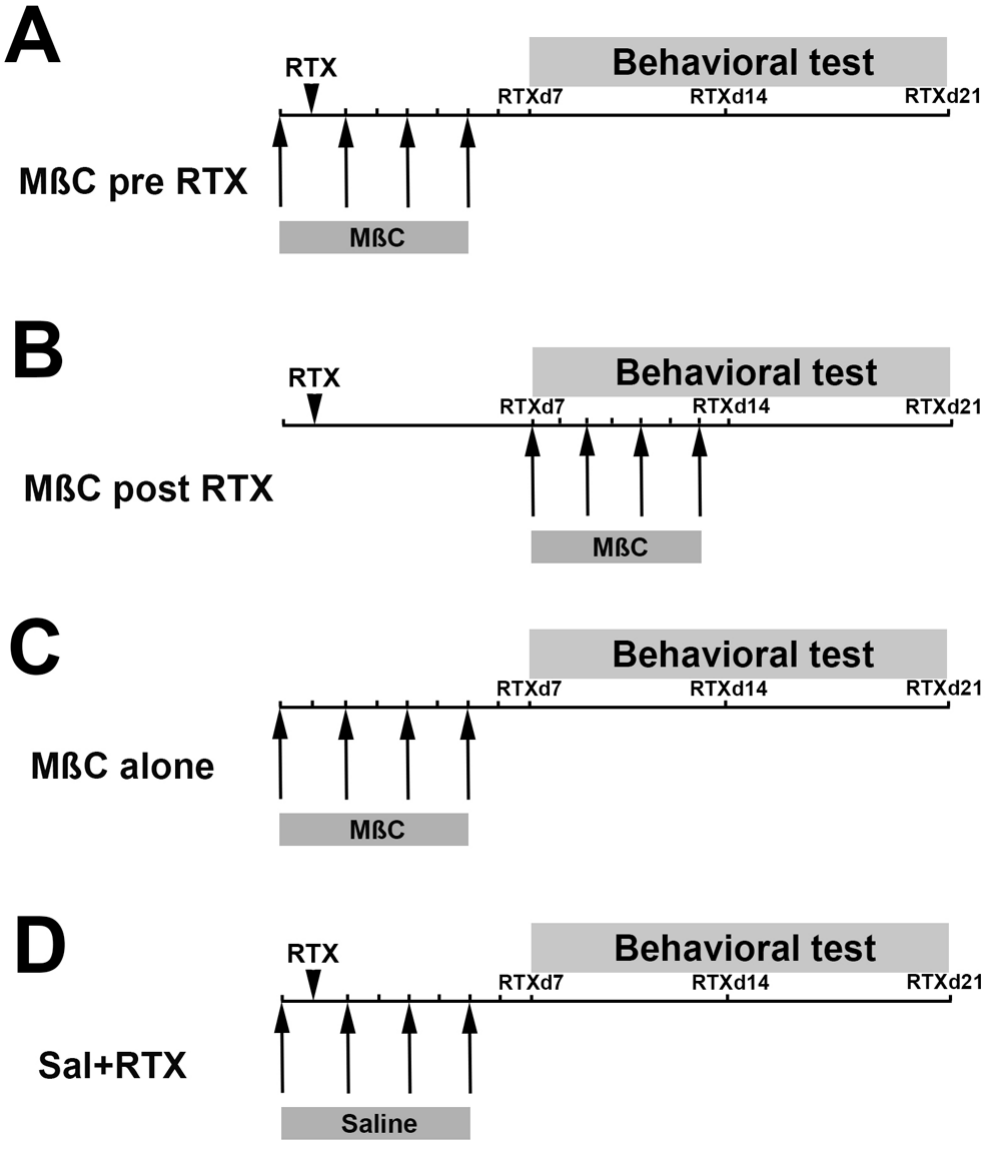
**Schedules of methyl-β-cyclodextrin (MβC) administration in resiniferatoxin (RTX) neuropathy.** MβC was administered in four doses through an intrathecal lumbar puncture (1 µg/5 µl, cumulative 4 µg per mouse) in different administered protocols described in the Materials and Methods. The arrowhead indicates RTX administration, and the arrows represent the time point of MβC administration. Behavior was tested at day 7 (RTXd7), RTXd14 and RTXd21 after RTX neuropathy. Schedules of MβC administration in RTX neuropathy for two protocols: (A) RTX mice that received MβC at RTXd0, RTXd1, RTXd3 and RTXd5 were assigned to the MβC pre-RTX group; (B) RTX mice that received MβC administered at RTXd7, RTXd9, RTXd11 and RTXd13 after RTX neuropathy were assigned to the MβC post-RTX group. The two control groups were as following: (C) naïve mice that received MβC were a positive control (MβC alone group), and (D) RTX mice that received saline were the negative control (Sal+RTX group).

We conducted immunohistochemistry on the dorsal root ganglion (DRG) neurons and performed pharmacological interventions with methyl-β-cyclodextrin (MβC) to deplete membrane cholesterol contents in RTX neuropathy (Fig. 1). This study demonstrated that MβC-mediated cholesterol depletion preserved PAP-mediated antinociception and that depletion of TRPV1(+) neurons mediated nerve degeneration leading to pain hypersensitivity through downregulation of the PAP antinociceptive effect.

## RESULTS

### Profiling membrane compositions in RTX neuropathy

**Fig. 2.**
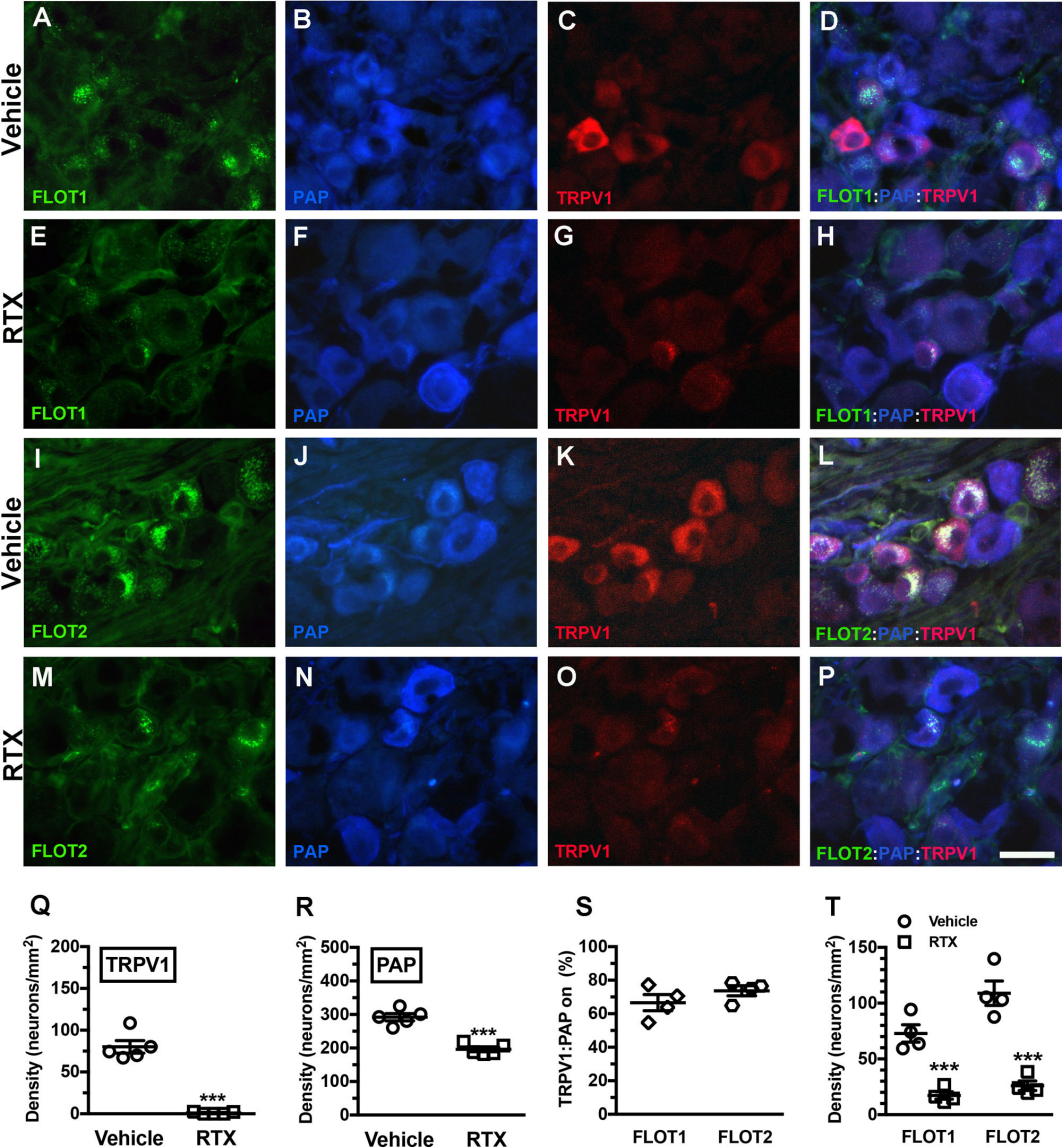
**Changes in prostatic acid phosphatase (PAP) and transient receptor potential vanilloid subtype 1 (TRPV1) colocalization with flotillins in RTX neuropathy.** (A–H) Triple-labeling immunofluorescent staining performed using anti-flotillin 1 (FLOT1; A,D,E,H), anti-PAP (B,D,F,H), and anti-TRPV1 antisera (C,D,G,H) on the dorsal root ganglia sections of the vehicle (A–D) and RTX groups (E–H). (D,H) FLOT1, PAP and TRPV1 merged for analyzing colocalized patterns on the vehicle (D) and RTX groups (H). (I–P) Analysis of the colocalization of flotillin 2 (FLOT2)/PAP/TRPV1. (Q,R) Changes in TRPV1(+) (Q) and PAP(+) neuronal densities (R) on the vehicle (open circle) and RTX groups (open square) according to Fig. 2A–P. (S) Ratio of TRPV1(+):PAP(+) neurons colocalized on FLOT1(+) (open diamond, *n*=5) and FLOT2(+) neurons (open hexagon, *n*=5) according to Fig. 2A–D and I–L. TRPV1 and PAP are highly coexistent on the same membrane microdomain. (T) Depletion of FLOT1(+) and FLOT2(+) neurons in the vehicle (open circle, *n*=5) and RTX groups (open square, *n*=5). ***P<0.001. Scale bar: 25 µm.

Triple-labeling immunofluorescence staining was performed to assess the colocalization of TRPV1 and PAP with FLOT1 (Fig. 2A,D,E,H) and FLOT2 (Fig. 2I,L,M,P), which reside in part in microdomains. The results showed that 67% of TRPV1 and PAP colocalized with FLOT1 and 74% with FLOT2 (Fig. 2S). As in our previous findings, TRPV1(+) neurons were completely depleted (80.1±16.7 versus 1.0±1.3 neurons/mm^2^; *P*<0.001) (Fig. 2Q) (Hsieh et al., 2012a), and mild depletion was present in PAP(+) neurons (291.9±24.0 versus 196.2±16.3 neurons/mm^2^; *P*<0.001) (Fig. 2R) (Wu et al., 2016) after RTX neuropathy due to PAP and TRPV1 colocalization. Accordingly, the FLOT1(+) neurons demonstrated a 75% reduction compared with the vehicle group (70.0±16.7 versus 17.2±6.7 neurons/mm^2^; *P*<0.001) due to coexpression with TRPV1, and the FLOT2(+) neuronal profiles had patterns similar to the FLOT1 profiles (108.9±22.0 versus 26.0±8.5 neurons/mm^2^; *P*<0.001; Fig. 2T).

We also examined the colocalization of TRPV1 and A1R with FLOT1 and FLOT2 (Fig. S2). TRPV1 and A1R colocalized with FLOT1 and FLOT2 (Fig. S2A–H,Q) to a higher level than A1R and PAP (Fig. S2I–P,R).

### Effect of MβC treatment on thermal and mechanical neuropathy

**Fig. 3.**
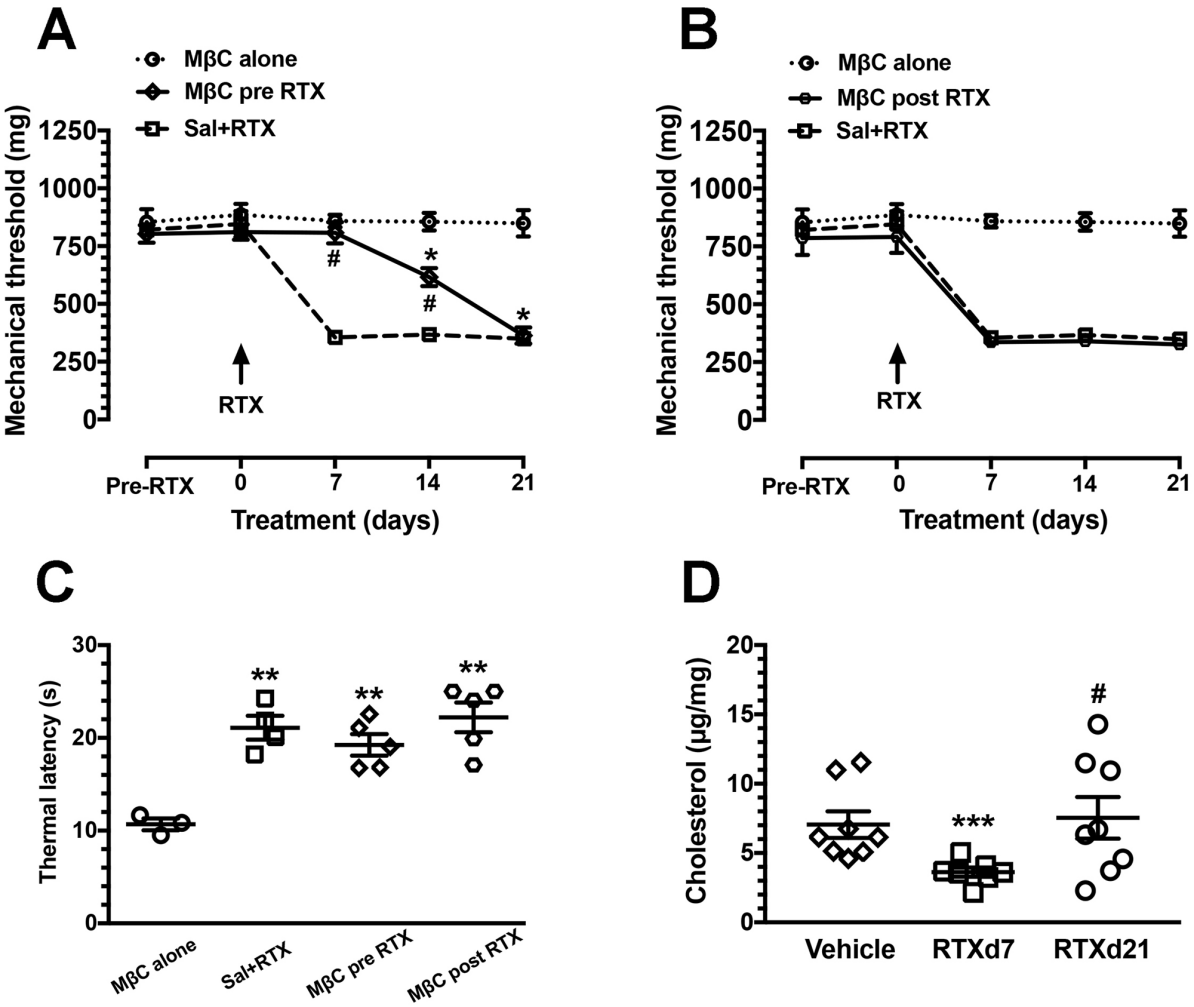
**Effect of membrane cholesterol depletion by MβC on neuropathic manifestation in RTX neuropathy.** (A,B) Effect of membrane cholesterol depletion in the mechanical allodynia of RTX neuropathy. Membrane integrity was disrupted by four doses of MβC (1 µg/day, cumulative 4 µg per mouse) through an intrathecal lumbar puncture, and the changes in mechanical threshold were evaluated at day 7 of RTX neuropathy (RTXd7), RTXd14 and RTXd21 as described in the Materials and Methods. Cholesterol depletion by MβC delivered before RTX neuropathy (MβC pre-RTX group, A) delayed the onset of mechanical allodynia, but MβC had no effect on mice in which RTX neuropathy was established (MβC post-RTX, B). For MβC pre-RTX, MβC was delivered at RTXd0, RTXd1, RTXd3 and RTXd5; for Sal+RTX, animals with RTX neuropathy received additional saline as control; for MβC alone, naïve mice received MβC as per the schedule of the MβC pre-RTX group; for the MβC post-RTX, MβC was delivered at RTXd7, RTXd9, RTXd11 and RTXd13. Arrows indicate the time points of RTX administration. *P<0.05: pairing repeated measures analysis of variance (ANOVA) followed Tukey *post hoc* test comparing before and after of MβC administration in the MβC pre-RTX group. ^#^*P<*0.05: nonpairing repeated measures ANOVA followed Tukey *post hoc* test between the MβC pre-RTX and Sal+RTX groups. (C) Thermal responses after RTX neuropathy were evaluated using the hot plate test at 52°C as described in the Materials and Methods. Thermal hypoalgesia was induced by RTX and MβC had no effect on the thermal responses. **P<0.01 Sal+RTX, MβC pre-RTX, and MβC post-RTX groups compared with the MβC alone group. (D) Total cholesterol content of the DRG tissues was evaluated using a cholesterol assay kit following the manufacturer's instructions in the vehicle group (open diamond, *n*=8) and at d7 (open square, *n*=7) and d21 (open circle, *n*=8) in the MβC alone group. This graph indicates that MβC depleted membrane cholesterol and resulted in disrupting the membrane integrity. ***P<0.001: d7 of the MβC alone group compared with the vehicle group. ^#^*P*<0.05: d7 compared with d21 of the MβC alone group.

We further investigated the functional significance of MβC treatment on neuropathic manifestations (Fig. 3). In the Sal+RTX group, as in our previous report on RTX neuropathy (Hsieh et al., 2012a; Wu et al., 2016), the mechanical thresholds were reduced at RTXd7 (839.1±216.6 versus 382.9±36.0 mg, *P*<0.05) through to RTXd21 (297.6±45.1 mg, *P*<0.05), and these mechanical allodynia were not observed in the MβC alone group, which was similar to our previously reported vehicle treatment (Fig. 3A,B) (Hsieh et al., 2012a; Wu et al., 2016). After cholesterol depletion by MβC (MβC pre-RTX group), mechanical allodynia induction was prevented at RTXd7 (781.0±181.5 mg, *P*>0.05), and these effects were gradually reduced from RTXd14 (619.1±114.5 mg, *P*<0.05) to RTXd21 (363.5±93.6 mg, *P*<0.001) (Fig. 3A). In the MβC post-RTX group, however, MβC had no effect once mechanical allodynia had developed at RTXd7, which was similar to the effect seen in the Sal+RTX group (336.7±21.7 versus 382.9±36.0 mg, *P*>0.05) (Fig. 3B). Notably, MβC failed to reverse thermal hypoalgesia in RTX neuropathy (Fig. 3C). The effect of MβC on pain transduction may be attributable to the disruption of membrane integrity by the depletion of membrane cholesterol components (Fig. 3D) (Ando et al., 2010).

### Profile changes of PAP and TRPV1 caused by the depletion of membrane cholesterol

**Fig. 4.**
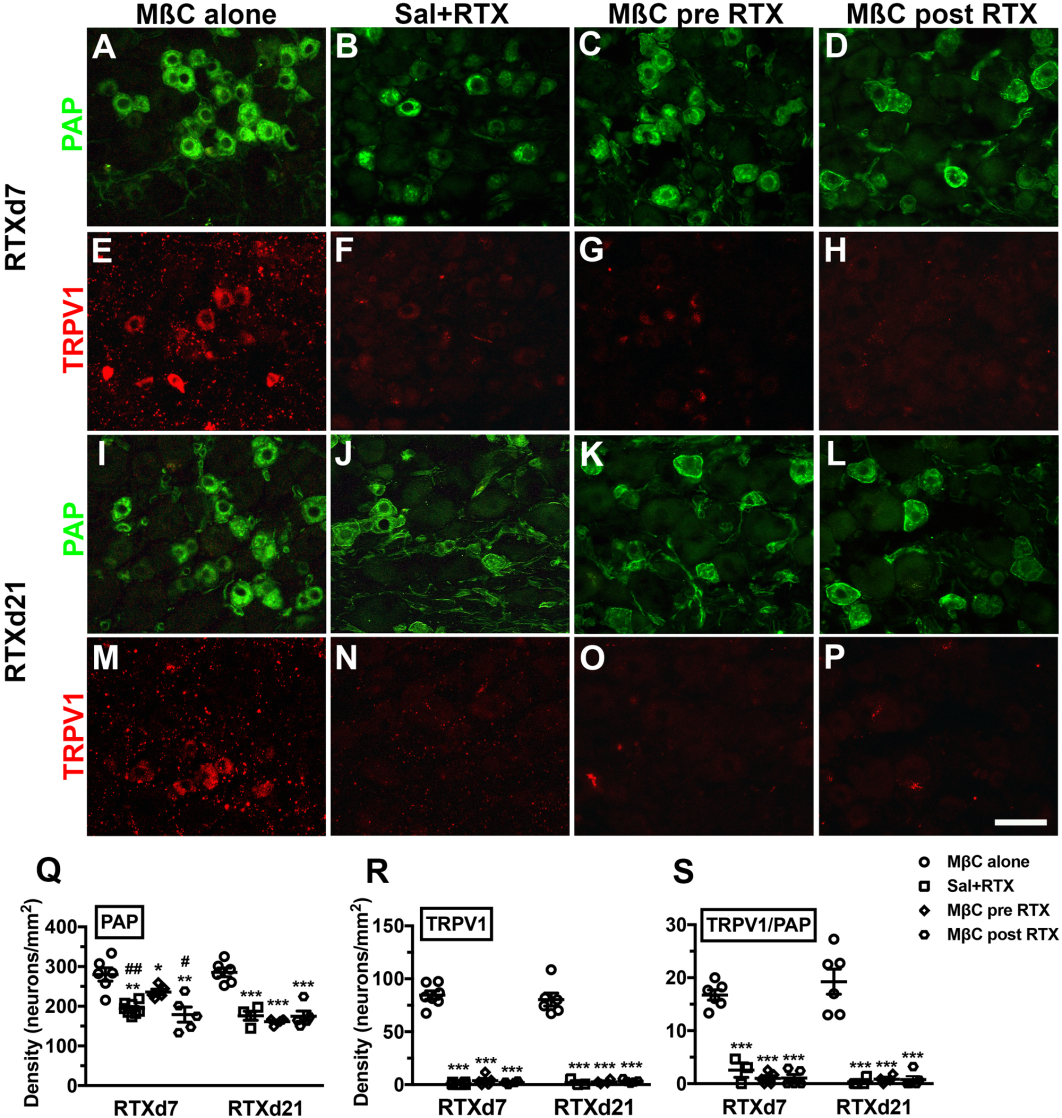
**Profile changes in PAP and TRPV1 in the dorsal root ganglion neurons of RTX neuropathy.** (A–H) Double-labeling immunofluorescent staining was performed with anti-PAP (A–D in green) and anti-TRPV1 (E–H in red) antisera at day 7 after RTX neuropathy (RTXd7) in the MβC alone (A,E), Sal+RTX (B,F), MβC pre-RTX (C,G) and MβC post-RTX groups (D,H). The PAP and TRPV1 profiles revealed different patterns for each group. (I–P) Similar approaches were applied to investigate the profiles of PAP and TRPV1 at RTXd21. (Q–S) Changes in PAP(+) (Q) and TRPV1(+) neuronal densities (R), and the colocalization ratios of TRPV1(+)/PAP(+) neurons (S) at RTXd7 and RTXd21 in the MβC alone (open circle, *n*=6), Sal+RTX (open square, *n*=6), MβC pre-RTX (open diamond, *n*=6), and MβC post-RTX group (open hexagon, *n*=6) according to A–P. For MβC pre-RTX, MβC was delivered at RTXd0, RTXd1, RTXd3 and RTXd5; for Sal+RTX, animals with RTX neuropathy received additional saline as control; for MβC alone, naïve mice received MβC as per the schedule of the MβC pre-RTX group; for the MβC post-RTX, MβC was delivered at RTXd7, RTXd9, RTXd11 and RTXd13. Scale bar: 50 µm. *P<0.05, **P<0.01, ***P<0.001: Sal+RTX, MβC pre-RTX and MβC post-RTX groups compared with the MβC alone group, respectively. ^#^*P*<0.05, ^##^*P*<0.01: Sal+RTX and MβC post-RTX groups compared with the MβC pre-RTX group, respectively.

We subsequently investigated the expression profiles of PAP and TRPV1 after cholesterol depletion by MβC treatment (Fig. 4). At RTXd7, the effect of MβC on the PAP(+) neuronal profiles were distinct; for example, the PAP(+) neurons were depleted in the Sal+RTX and MβC post-RTX groups, whereas only mild PAP depletion, occurred in the MβC pre-RTX group (Fig. 4A–D). Quantitatively, PAP(+) neuronal densities decreased approximately 30% in the Sal+RTX group (194.2±15.2 versus 280.1±40.7 neurons/mm^2^; *P*=0.0002) and 36% in the MβC post-RTX group (179.3±8.0 neurons/mm^2^; *P*=0.0091) compared with the MβC alone group. In the MβC pre-RTX group, PAP(+) neuronal density decreased approximately 16% (235.8±15.9 neurons/mm^2^), and these neuronal densities were higher than the neuronal densities in the Sal+RTX (*P*=0.015) and MβC post-RTX groups (*P*=0.016) (Fig. 4Q). However, at RTXd21, the PAP(+) neuronal densities in the Sal+RTX, MβC pre-RTX, and MβC post-RTX groups were identical and significantly lower than those in the MβC alone group (Fig. 4I–L,Q).

The TRPV1(+) neuronal densities were completely depleted in the Sal+RTX, MβC pre-RTX, and MβC post-RTX groups (Fig. 4R) [i.e. low ratios in TRPV1(+)/PAP(+) neurons compared with the MβC alone group, Fig. 4S]. Collectively, RTX neurotoxicity to PAP was compromised by cholesterol depletion by MβC, which might be due to an effect on the integrity of membrane microdomains.

### PI(4,5)P2 hydrolysis after disruption of membrane integrity

**Fig. 5.**
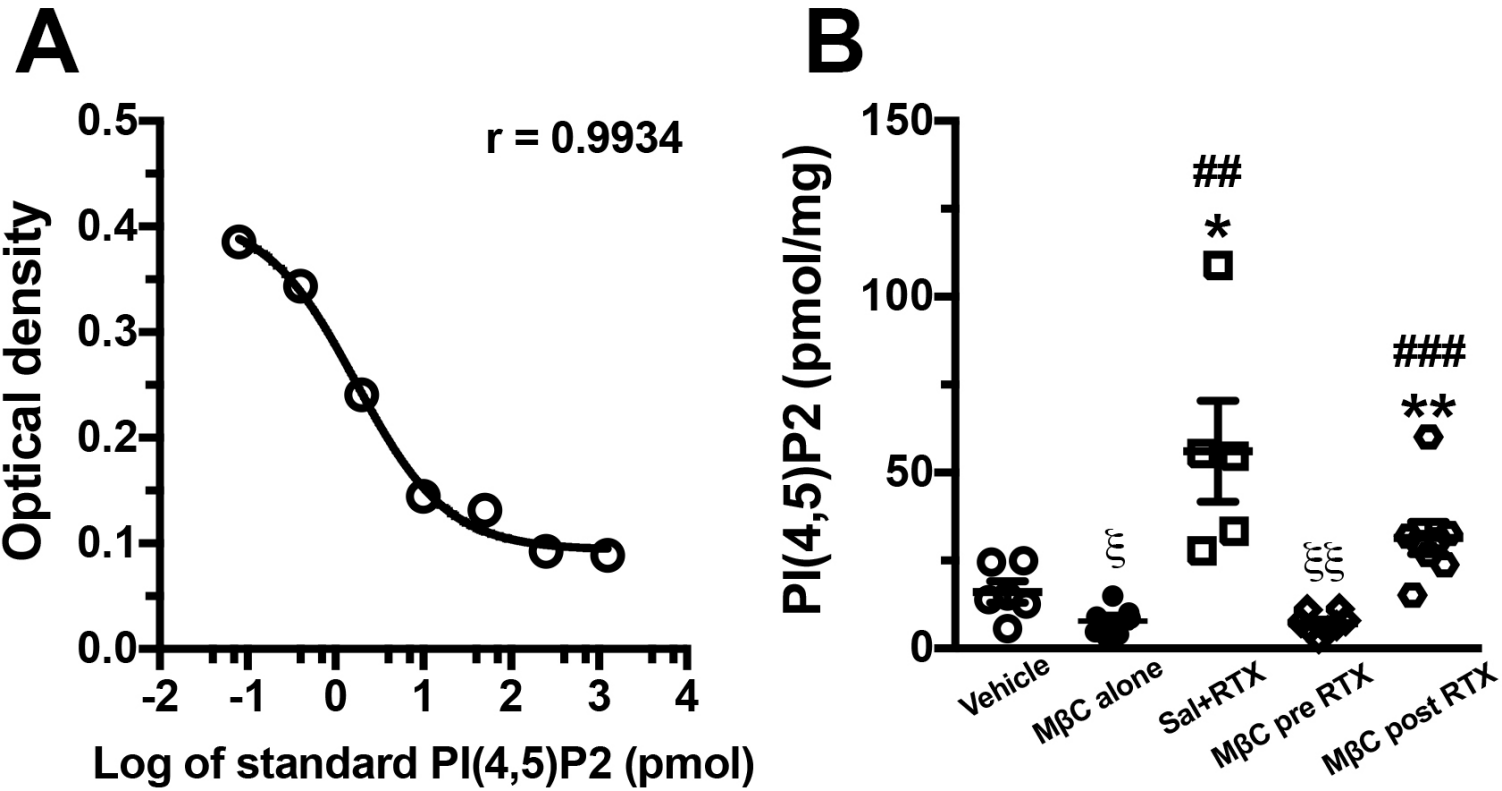
**Changes in phosphatidylinositol 4,5-bisphosphate [PI(4,5)P2] content after membrane cholesterol depletion by MβC in RTX neuropathy.** PI(4,5)P2 content was determined through the enzyme-linked immunosorbent assay following a nonlinear fit regression of the PI(4,5)P2 standard concentration as described in the Materials and Methods. (A) Nonlinear fit regression revealed that the PI(4,5)P2 content was fitted to the optical densities of the standard curve (*P*=0.9934) determined using a 450 nm wavelength. (B) Changes in PI(4,5)P2 content in the vehicle (open circle, *n*=6), MβC alone (filled circle, *n*=5), Sal+RTX (open square, *n*=5), MβC pre-RTX (open diamond, *n*=5), and MβC post-RTX groups (open hexagon, *n*=8). For the MβC pre-RTX group, MβC was delivered at RTXd0, RTXd1, RTXd3 and RTXd5. For the Sal+RTX group, animals with RTX neuropathy received additional saline as control. For the MβC alone group, naïve mice received MβC as per the schedule of the MβC pre-RTX group. For the MβC post-RTX group, MβC was delivered at RTXd7, RTXd9, RTXd11 and RTXd13. *P<0.05, **P<0.01: Sal+RTX, MβC pre-RTX and MβC post-RTX groups compared with the MβC alone group, respectively. ^##^*P*<0.01, ^###^*P*<0.001: Sal+RTX and MβC post-RTX groups compared with the MβC pre-RTX group, respectively. ^ξ^*P<*0.05, ^ξξ^*P*<0.01: MβC alone and MβC pre-RTX groups compared with the vehicle group, respectively.

PAP mediates PI(4,5)P2 hydrolysis, which sequentially reduces TRPV1-mediated nociception (Sowa et al., 2010). PI(4,5)P2 is a membrane phospholipid; consequently, we also investigated whether PI(4,5)P2 content is affected by MβC treatment. The PI(4,5)P2 content from DRG extraction was determined by a standard curve of PI(4,5)P2 standard concentration, which followed nonlinear fit regression (r=0.9934, Fig. 5A). Notably, the changes in PI(4,5)P2 content was inverse to the changes in PAP(+) neuronal densities, i.e., PI(4,5)P2 contents in Sal+RTX (47.9±37.1 pmol/mg) and MβC post-RTX (31.4±13.0 pmol/mg) group were markedly higher than those in the MβC alone (7.8±4.1 pmol/mg) and MβC pre-RTX (7.2±2.9 pmol/mg) groups (Fig. 5B versus Fig. 4Q). Notably, PI(4,5)P2 contents in the MβC alone (*P*=0.014) and MβC pre-RTX (*P*=0.005) groups were significantly lower than that in the vehicle group (16.1±7.5 pmol/mg).

### Treatment with MβC had no effect on the prevention of neuronal injury

**Fig. 6.**
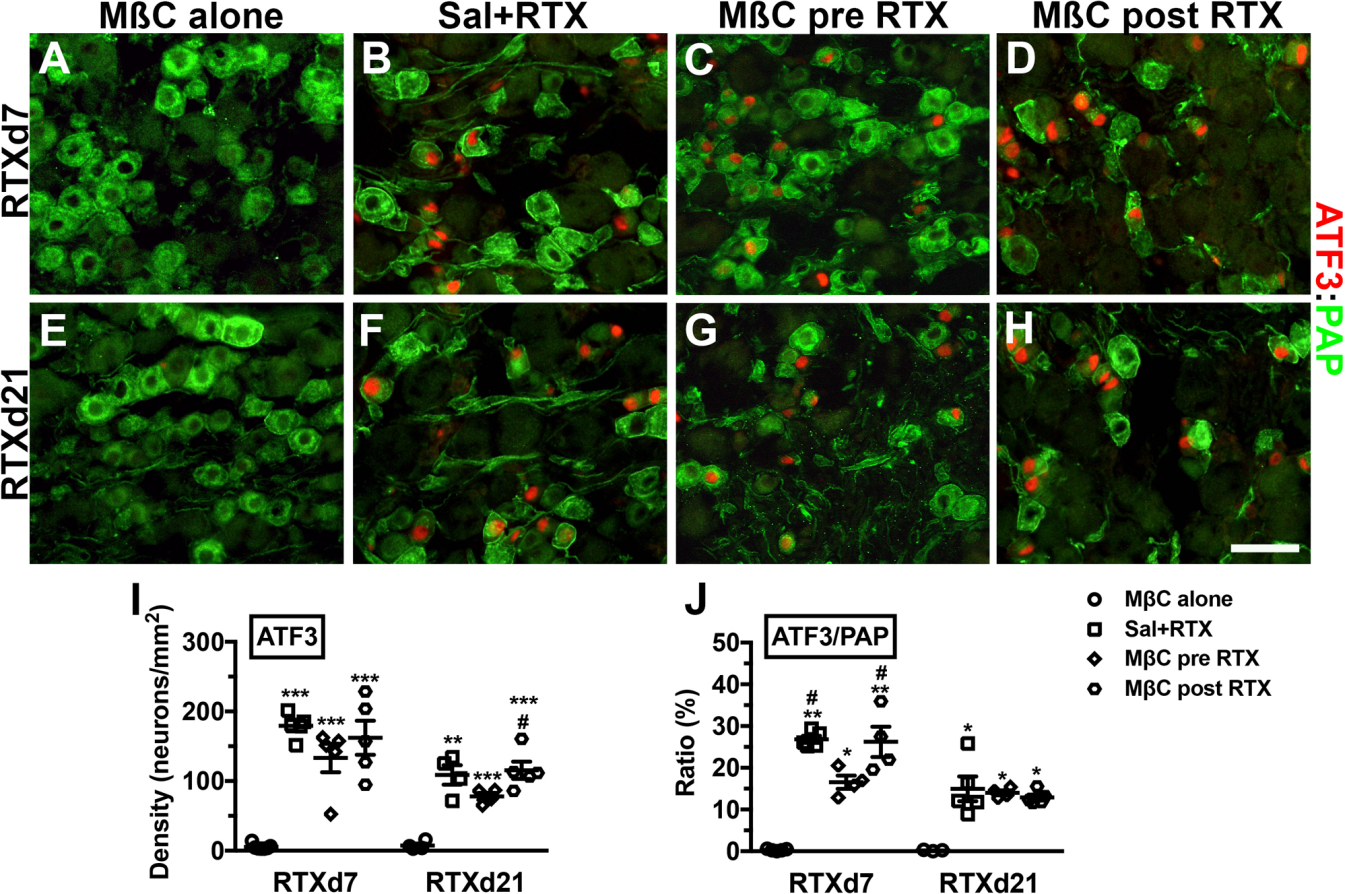
**Effect of membrane cholesterol depletion by MβC on the expression profiles of PAP and activating transcription factor-3 (ATF3) in the dorsal root ganglion neurons of RTX neuropathy.** (A–D) Double-labeling immunofluorescent staining was performed using anti-PAP (A–D in green) and anti-ATF3 (A–D in red) at day 7 after RTX neuropathy (RTXd7) in the MβC alone (A), Sal+RTX (B), MβC pre-RTX (C) and MβC post-RTX groups (D). ATF3 was upregulated in the Sal+RTX, MβC pre-RTX, and MβC post-RTX groups but not in the MβC alone group. (E–H) Similar approaches were used to investigate the profiles of ATF3 and PAP at RTXd21. *P<0.05, **P<0.01, ***P<0.001: the Sal+RTX, MβC pre-RTX, and MβC post-RTX groups compared with the MβC alone group, respectively. ^#^*P*<0.05, ^##^*P*<0.01: the Sal+RTX and MβC post-RTX groups compared with the MβC pre-RTX group. For MβC pre-RTX, MβC was delivered at RTXd0, RTXd1, RTXd3 and RTXd5; for Sal+RTX, animals with RTX neuropathy received additional saline as control; for MβC alone, naïve mice received MβC as per the schedule of the MβC pre-RTX group; for the MβC post-RTX, MβC was delivered at RTXd7, RTXd9, RTXd11 and RTXd13. Scale bar: 50 µm.

We previously demonstrated that RTX induced ATF3 upregulation, reflecting neuronal injury (Hsieh et al., 2012a; Wu et al., 2016) and that the ratios of ATF3(+)/PAP(+) neurons correlated with the degree of mechanical allodynia (Wu et al., 2016). In the current study, we investigated whether RTX neuropathy with MβC treatment affected ATF3 profiles with double labeling of ATF3(+)/PAP(+) neurons (Fig. 6). Notably, the Sal+RTX (179.5±18.0 neurons/mm^2^, *P*<0.001), MβC pre-RTX (133.3±45.8 neurons/mm^2^, *P*<0.001), and MβC post-RTX (162.3±54.7 neurons/mm^2^, *P*<0.001) groups had marked ATF3 upregulation compared with the MβC alone group (5.5±4.0 neurons/mm^2^) at RTXd7 (Fig. 6A–D,I). At RTXd21, each group had similar ATF3 profiles, although the ATF3(+) neurons were decreased (Fig. 6E–H,I). This upregulation of ATF3 on PAP(+) neurons, however, was reduced [i.e. the ratio of ATF3(+)/PAP(+) neurons in the MβC pre-RTX group at RTXd7 was significantly lower], indicating lower injury degree of PAP(+) neurons (Fig. 6J versus 4Q).

### Treatment with MβC had no effect on CGRP(+) IENFs

**Fig. 7.**
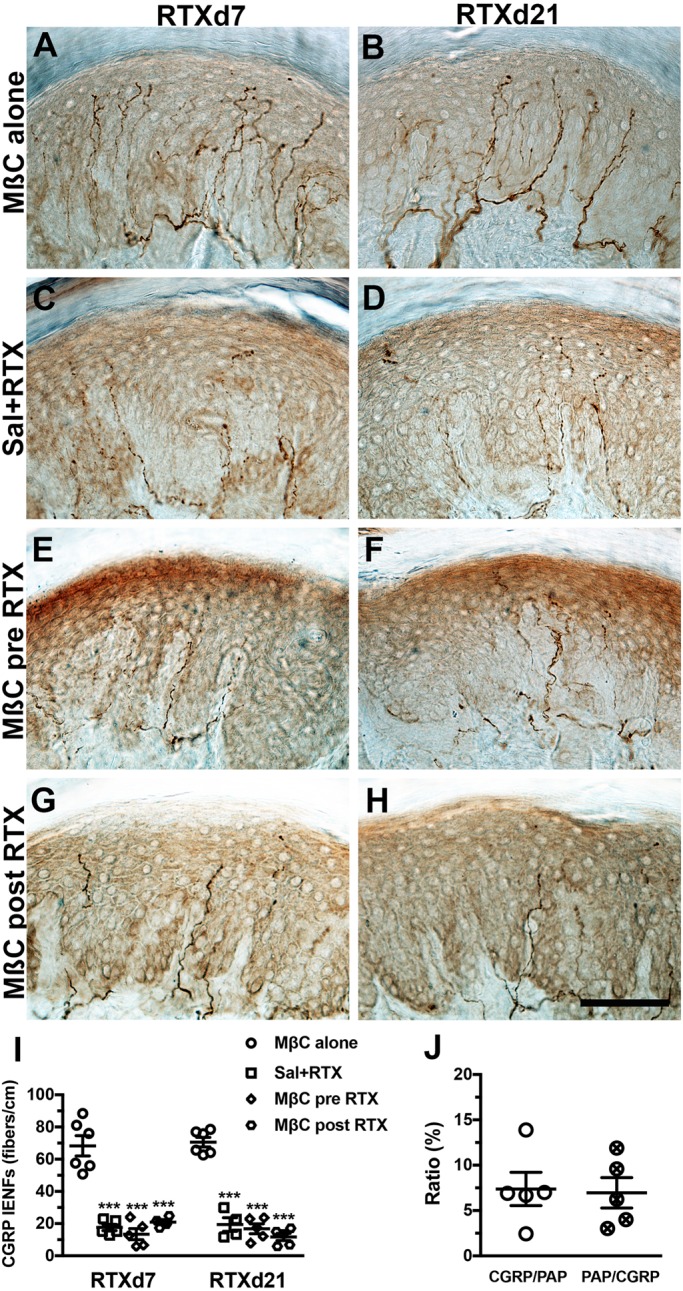
**MβC had no effect on calcitonin gene-related peptide (CGRP)(+) intraepidermal nerve fibers (IENFs) in RTX neuropathy.** (A–H) CGRP(+) IENFs with anti-CGRP antisera at day 7 after RTX neuropathy (RTXd7; A,C,E,G) and RTXd21 (B,D,F,H), respectively, in the MβC alone (A,B), Sal+RTX (C,D), MβC pre-RTX (E,F) and MβC post-RTX (G,H) groups. (I) Quantification of CGRP(+) IENFs at RTXd7 and RTXd21 in the MβC alone (open circle, *n*=6), Sal+RTX (open square, *n*=5), MβC pre-RTX (open diamond, *n*=5), and MβC post-RTX (open hexagon, *n*=5) groups according to A–H. (J) Colocalized ratio of CGRP/PAP (open circle) and PAP/CGRP (cross circle) quantified according to Fig. S4A–C. Scale bar: 100 µm. ***P<0.001: Sal+RTX, MβC pre-RTX and MβC post-RTX groups compared with the MβC alone group.

CGRP(+) IENFs compensate for the thermal withdrawal after TRPV1 depletion in RTX neuropathy (Hsieh et al., 2012b). The present study demonstrated that MβC-induced microdomain disruption in RTX neuropathy had no effect on the profiles of CGRP(+) IENFs (Fig. 7); CGRP(+) IENFs were depleted in the Sal+RTX (68.3±15.5 versus 17.6±4.5 fibers/cm, *P*<0.001), MβC pre-RTX (13.4±7.7 fibers/cm, *P*<0.001), and MβC post-RTX (20.9±3.2 fibers/cm, *P*<0.001) groups whereas no depletion occurred in the MβC-alone group at RTXd7. These groups had similar patterns at RTXd21 (Fig. 7I). These distinct outcomes on PAP and CGRP resulted from the limited colocalization of PAP and CGRP (Fig. S3), with a colocalization ratio of approximately 7% (Fig. 7J).

## DISCUSSION

This study demonstrated that membrane integrity is in involved in RTX neuropathy through two mechanisms: (1) the prevention of allodynic pain by MβC-mediated cholesterol depletion and (2) the recurrence of pain caused by the dysfunction of PAP-mediated antinociception related to TRPV1(+) neuron depletion–mediated neurodegeneration.

### Integrity of membrane microdomains as functional units responsible for neuropathology and nociceptive transduction

**Fig. 8.**
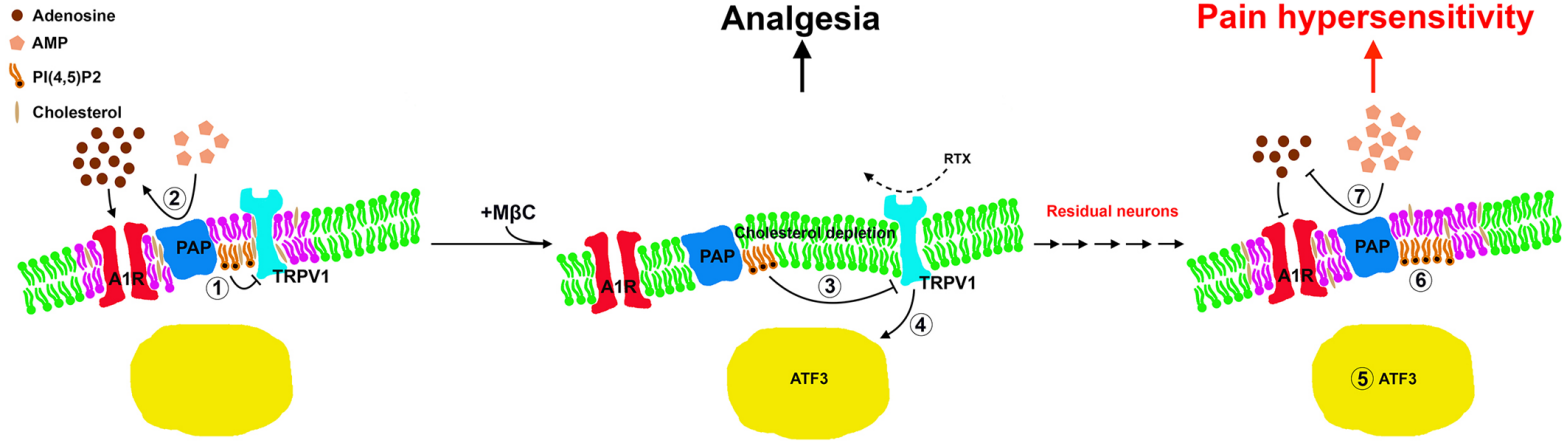
**Antinociceptive dysfunction by disrupting of cholesterol-rich membrane microdomains with MβC in RTX neuropathy.** Cholesterol-rich membrane microdomains act as novel units for pain modulation in RTX neuropathy through the following mechanisms. (Left panel) In a silent neuron, TRPV1 colocalizes with adenosine signaling molecules such as PAP and A1R in a specific cholesterol-rich membrane microdomains (labeled in purple; rest of the plasma membrane labeled in green), resulting in (1) hydrolyzing PI(4,5)P2 that consequently reduces TRPV1 sensitization and (2) AMP hydrolyzing to adenosine, resulting in antinociception. (Middle panel) (3) At the acute stage of RTX neuropathy, MβC disrupted the microdomain by depleting cholesterol, which preserves PAP-hydrolyzed PI(4,5)P2 and antinociception despite (4) TRPV1 sensitization and ATF3 upregulation following RTX administration. (Right panel) At the chronic stage, depletion of TRPV1(+) neurons is mildly associated with PAP(+) and A1R(+) neuron depletion; moreover, (5) the residual A1R(+) and PAP(+) neurons are associated with ATF3 upregulation, (6) suppression of PI(4,5)P2 hydrolysis, and (7) reduced availability of adenosine (Kan et al., 2018), which consequently induces pain hypersensitivity.

Membrane microdomains contain pain-sensing receptors that modulate pain transduction (Marchenkova et al., 2016); consequently, the disruption of microdomains at the periphery of sensory neurons relieves primary chronic pain (Ferrari and Levine, 2015). The current study's findings provide further evidence that disruption of the membrane microdomains of sensory neurons prevents nociceptive development by preserving PAP-mediated antinociception. Changes in molecular distribution correlate with peripheral neuropathy (Gambert et al., 2017; Lee et al., 2014), and studies have focused on manipulating and altering microdomain structures to confirm their roles in pain modulation. The current study provides pathological and biochemical evidence of the role of membrane composition in pain modulation in addition to the role of transient cholesterol depletion. In particular, PAP-mediated antinociception caused by maintaining PI(4,5)P2 hydrolysis and TRPV1 depletion were correlated with pain development after RTX neuropathy. The lipid composition of microdomains is complex (Saghy et al., 2015), but one important component appears to be cholesterol, which can be depleted by MβC. Cholesterol has received increasing attention for its relation to nociceptive modulation (Gnanasekaran et al., 2011; Kumari et al., 2015; Licon et al., 2015; Saghy et al., 2015). This study determined that cholesterol on membrane microdomains was transiently depleted by MβC, implying that TRPV1 and PAP are located in cholesterol-rich microdomains. TRPV1 and PAP colocalize with FLOT1 and FLOT2, suggesting that they are involved in intracellular signaling transduction during normal neurophysiology (Lee et al., 2014). The molecular intervention between TRPV1 and PAP involves a PI(4,5)P2 signal. This article provides the first pathological and functional evidence demonstrating the correlation between PI(4,5)P2 hydrolysis and membrane cholesterol content in addition to the signal-regulation of TRP channel-dependent activity (Certal et al., 2017). Notably, FLOT1 and FLOT2 were markedly depleted in RTX neuropathy associated with TRPV1(+) neuronal depletion. Taken together with our previous study (Wu et al., 2016), these results suggest that microdomains on sensory neurons act as functional units for pain transduction in two stages: (1) in the acute stages of small-fiber neuropathy, disruption of microdomains containing TRPV1 and adenosine signaling molecules prevents pain transmission and (2) in the chronic stages, the degree of neuronal injury is the critical factor for pain hypersensitivity in addition to the molecular interaction within microdomains (Fig. 8). However, the signal cascade linkages from cholesterol depletion to activation of the nociceptive messengers in irritated neuronal soma remain elusive and require further investigation.

### Molecular significance of TRPV1 and PAP in the same microdomain mediating pain development

TRPV1 is considered a thermosensor in TRPV1-depletion dependent thermal analgesia. RTX is an ultrapotent capsaicin analog that sensitizes TRPV1 and our previous studies have characterized the neuropathic manifestation of RTX neuropathy model. For example, RTX induced thermal hypoalgesia through TRPV1(+) neuronal depletion and IENF degeneration (Hsieh et al., 2008; Hsieh et al., 2012b), which coincided with mechanical allodynia caused by the enhancement of P2X3 purinergic signaling (Hsieh et al., 2012a) and injury of PAP(+) neurons with an imbalance of adenosine signaling (Kan et al., 2018; Wu et al., 2016). Paradoxically, RTX-induced mechanical allodynia has not been observed with alternative routes of administration (Hockman et al., 2018; Sapio et al., 2018). Accordingly, RTX administered intraperitoneally was systemically but not locally effective in a dose-dependent manner (Lee et al., 2018). Indeed, RTX systemic neurotoxicity has also been demonstrated to induce mechanical allodynia in other species (Pan et al., 2003), and this RTX neuropathy model could mimic distinct neuropathic manifestations, such as those of diabetes patients, in clinics (Hsieh et al., 2018). Furthermore, the current study demonstrated that TRPV1 and PAP colocalize with FLOT1 and FLOT2; accordingly, TRPV1 mediates nociceptive transduction through PI(4,5)P2 signaling between TRPV1 and PAP in regions containing these flotillins. MβC disrupted membrane integrity through cholesterol depletion, which maintained PI(4,5)P2 hydrolysis, thereby preventing pain development.

TRPV1 depletion in RTX neuropathy paralleled the development of pain hypersensitivity, decrease in PAP(+) neuronal densities, and PI(4,5)P2 hydrolysis. Lower PAP(+) neuronal densities resulted in higher PI(4,5)P2 availability, which acted as an agonist of TRPV1 that modulated TRPV1 activity (Bevan et al., 2014; Poblete et al., 2015). Although PI(4,5)P2 was more readily available following RTX neuropathy, it demonstrated lower efficacy in TRPV1 sensitization due to TRPV1 depletion. Greater PI(4,5)P2 availability may have molecular significance. We previously demonstrated that the enhancement of P2X3 purinergic signaling correlates linearly to the degree of mechanical allodynia (Hsieh et al., 2012a). Depletion of P2X3 by RTX was limited because of low colocalized ratios with TRPV1(+) neurons (Hsieh et al., 2012a); in fact, P2X3 may act as the downstream molecule of PI(4,5)P2 (Mo et al., 2009; Mo et al., 2013). Moreover, PI(4,5)P2-modulated P2X3 was suggested through autocrine signaling due to the high colocalization of PAP and P2X3 in DRG neurons (Wu et al., 2016).

### Clinical implications of lipid-derived components in microdomains for pain management

This study's mouse model of RTX neuropathy mimicked the characteristics of patients with small-fiber neuropathy (i.e. skin denervation and sensation disorders). Most studies have focused on investigating the responses and contributions of sensitized small-diameter nociceptors after those nociceptors suffer injury and irritation (Hsieh et al., 2012a; Orstavik et al., 2006; Serra et al., 2014). For example, an approximately 25% reduction of small-diameter neurons was noted, which particularly colocalized with TRPV1. Moreover, 45% of small-diameter neurons were injured during labeling by ATF3, and twice as many P2X3(+) neurons were sensitized on residual neurons (Hsieh et al., 2012a). These findings suggest that systemic RTX affects not only TRPV1(+) neurons but also other phenotypic small-diameter neurons. Therefore, this current study revealed an alternative mechanism for pain development, and manipulation and targeting of the microdomains that contain TRPV1 nociceptor should be the first line of pharmacotherapeutic treatment.

Specialized membrane microdomains contain cholesterol, sphingomyelin and gangliosides (Pike, 2003). In addition to the cholesterol depletion-prevented pain development documented in this report, TPRV1 activity is affected by altering ganglioside synthesis (Saghy et al., 2015; Santha et al., 2010; Szőke et al., 2010) and sphingomyelin inhibition (Saghy et al., 2015; Szőke et al., 2010). These lipid-derivative molecules, particularly sphingomyelin signaling, involve nociceptive modulation through activation of the p75 neurotrophin receptor (Khodorova et al., 2013; Khodorova et al., 2017). Moreover, some G protein-coupled receptors are localized in microdomains and participate in nociceptive transduction (Monastyrskaya et al., 2005; Nakagawa et al., 2017). Lipid metabolism disorders related to microdomain-attributed peripheral neuropathy are a potential target for pain management through the elimination of related lipid metabolic constituents (Gambert et al., 2017; Ogawa and Rasband, 2009; Smith et al., 2011).

This study systematically explored pain modulation from the role of microdomain integrity to postneuronal injury responses. Pain control targeting microdomains in the plasma membrane is a new direction for research that has received only limited attention. Notably, cholesterol in microdomains was determined to be sensitive to drug-induced microdomain disruption (Gomide et al., 2013), and multiple doses of MβC were required because of dynamic replenishment of cholesterol from intracellular stores (Mahammad and Parmryd, 2008), which means that the effect of MβC was transient (Fig. S3) (Ferrari and Levine, 2015). Contrarily, during the chronic stages of RTX neuropathy, TRPV1 depletion associated with neuronal injury triggered the dysfunction of PAP-mediated antinociception, consequently inducing pain transduction (Kan et al., 2018).

## MATERIALS AND METHODS

### Establishment of RTX neuropathy and disruption of membrane integrity

This study comprised two parts: (1) profiling and biochemically assessing a membrane molecular composition after RTX neuropathy and (2) functionally investigating the role of membrane integrity by depleting cholesterol through pharmacological interruption. In the RTX group, RTX neuropathy was induced using a single dose of RTX (Sigma-Aldrich; 50 µg/kg) solution through intraperitoneal (i.p.) injection in accordance with our previously established protocol (Hsieh et al., 2012a; Hsieh et al., 2008; Lin et al., 2013; Wu et al., 2016). Another group received a vehicle as a control (the vehicle group).

To investigate the role of membrane integrity in RTX neuropathy, we disrupted membrane cholesterol using MβC, an agent of cholesterol depletion (Ferrari and Levine, 2015) with a modified administered protocol (Fig. 1). Mice were randomly assigned to four groups: (1) MβC delivered before RTX treatment (RTXd0; all subsequent references abbreviated in the same format, RTXd*y*, where *y* stands for the number of days after RTX treatment), RTXd1, RTXd3 and RTXd5, which was the MβC pre-RTX group (Fig. 1A). MβC priority to RTX administration could deplete cholesterol and resulted in the disruption of membrane integrity, to investigate the interaction of TRPV1 with antinociceptive-related molecules. (2) MβC was delivered at RTXd7 following neuropathic manifestation (Hsieh et al., 2012a), and then at RTXd9, RTXd11 and RTXd13, which was the MβC post-RTX group (Fig. 1B). (3) Naïve mice received MβC in accordance with the same protocol as the MβC pre-RTX group except they did not receive RTX treatment, which was the positive control group (MβC alone group, Fig. 1C). (4) RTX mice received saline in accordance with the same protocol as the MβC pre-RTX group, which was the negative control group (Sal+RTX group, Fig. 1D). Cholesterol depletion was confirmed with the schedule of the MβC-alone group to examine the pathological and biochemical effect of MβC at RTXd7 and RTXd21 (Fig. S3). Behavior was evaluated at RTXd7, RTXd14 and RTXd21. After treatment, the mice were housed in plastic cages on a 12 h light/12 h dark cycle and were provided access to food and water *ad libitum*. All procedures were conducted in accordance with the ethical guidelines for laboratory animals (Zimmermann, 1983), and the protocol was approved by Kaohsiung Medical University. All experimental procedures were performed carefully, and every effort was made to minimize suffering.

### Animal behavior evaluation

The RTX mice appeared normal and exhibited no mechanical sensitivity during regular animal handling. Behavioral evaluations assessed thermal (i.e. the hot plate test) and mechanical (i.e. the von Frey filament test) responses.

#### Hot plate test

Mice were placed on a 52°C hot plate (IITC) enclosed in an acrylic cage. The withdrawal latencies of the hind paw to thermal stimulations were determined to an accuracy of 0.1 s. Each test session comprised three trials separated by 30 min intervals. The withdrawal criteria included shaking, licking or jumping on the hot plate. The mean latency was expressed as the threshold of an individual animal to the thermal stimulation.

#### von Frey filament test

The mechanical thresholds of the hind paw were assessed using an up-and-down method with various von Frey monofilament calibers (Somedic Sales AB). Several monofilaments were applied to the plantar region of the hind paw. If paw withdrawal occurred, a monofilament of a smaller caliber was applied. In the absence of paw withdrawal, a monofilament of a larger caliber was used. Four additional stimuli with monofilaments of different calibers based on the preceding responses were applied. The mechanical thresholds were calculated in accordance with a published formula (Chaplan et al., 1994).

### Immunofluorescent staining of DRG neurons

We conducted double- and triple-labeling immunofluorescent staining with various mixtures of primary antisera. All primary antisera in the current study were purchased commercially, and the following antisera were used: anti-PAP (chicken, 1:600, Aves Labs), anti-TRPV1 (goat, 1:100, Santa Cruz Biotechnology), anti-activating transcription factor 3 (ATF3, 1:100, Santa Cruz Biotechnology), anti-calcitonin gene-related peptide (CGRP, rabbit, 1:1000, Sigma-Aldrich), anti-flotillin 1 (FLOT1, mouse, 1:50, BD Transduction Laboratories) and anti-flotillin 2 (FLOT2, mouse, 1:50, BD Transduction Laboratories). The antisera used in this study were examined using the negative control group (Fig. S1). Animals were killed through intracardiac perfusion with 0.1 M phosphate buffer (PB) followed by 4% paraformaldehyde (4P) in 0.1 M PB. The fourth and fifth (L4/L5) lumbar DRG tissues were carefully removed after perfusion and postfixed in 4P for an additional 6 h. The DRG tissues were cryoprotected with 30% sucrose in 0.1 M PB overnight and cryosectioned using a cryostat (CM1850, Leica) at a thickness of 8 µm. To ensure adequate sampling, two ganglia (L4/L5) per mouse and five to eight sections per DRG tissue (at 80 µm intervals) were immunostained. The combinations of primary antisera included: (1) TRPV1:PAP, (2) ATF3:PAP, and (3) CGRP:PAP. The sections were incubated with a mixture of primary antiserum combinations at 4°C overnight, followed by incubation with either Texas Red (TR) or fluorescein isothiocyanate (FITC)-conjugated secondary antisera (1:100, Jackson ImmunoResearch), corresponding to the appropriate primary antisera for 1 h.

FLOT1 and FLOT2 are membrane-associated proteins that are considered microdomain markers. To investigate colocalization with FLOT1 and FLOT2, triple-labeling immunostaining of (1) TRPV1/PAP/FLOT1/2 and (2) the adenosine A1 receptor (A1R)/PAP/FLOT1/2 was conducted. The protocol was similar to a double-labeling study, and an additional corresponding aminomethylcoumarin acetate (AMCA)-conjugated secondary antiserum (1:100, Jackson ImmunoResearch) was used. Sections after immunofluorescent staining were mounted using Vectashield (Vector) for quantification.

### PI(4,5)P2 and cholesterol biochemical assay

#### PI(4,5)P2 measurement

To evaluate the PI(4,5)P2 change in RTX neuropathy, enzyme-linked immunosorbent assay (ELISA) was performed with the DRG tissues. The wet weight of the DRG tissues was determined and lipids were extracted. The PI(4,5)P2 contents were quantified using a PI(4,5)P2 Mass ELISA kit (Echelon Bioscience) in accordance with the manufacturer's instructions. The signal was recorded at 450 nm, and the PI(4,5)P2 contents were obtained from nonlinear fit standard curves and normalized by dividing the wet weight of the DRG tissues.

#### Cholesterol assay

Total cholesterol content of DRG tissues was evaluated using a cholesterol assay kit (BioVision). Briefly, cholesterol was extracted from DRG tissues per the manufacturer's instructions, and changes in cholesterol concentration after MβC treatment were determined. A colorimetric assay was conducted at an optical density of 570 nm to determine the content of cholesterol from a standard curve, which was normalized by dividing the wet weight of the DRG tissues.

### Drug preparation

RTX (Sigma-Aldrich) was dissolved in a vehicle (10% Tween 80 and 10% ethanol in normal saline) and aliquot at a working concentration (1 µg/10 µl) before being stored in a freezer at −20°C. MβC (Sigma-Aldrich) was prepared using 10% DMSO in saline and delivered in four doses (1 µg/5 µl per dose and a cumulative 4 µg per mouse) through intrathecal lumbar puncture (i.t.) route (Lin et al., 2013).

### Immunohistochemistry of CGRP(+) IENFs

Footpad tissues were cryosectioned with a sliding microtome at a thickness of 30 µm. To ensure adequate sampling, every third section for a total of six sections from each mouse was chosen and immunostained. Footpad skin was incubated using anti-CGRP (rabbit, 1:1500, Sigma-Aldrich) antiserum overnight at 4°C and rinsed in Tris buffer. Those sections were subsequently incubated with biotinylated goat anti-rabbit immunoglobulin G (Vector) for 1 h and avidin-biotin complex (Vector) for another hour. The reaction product was demonstrated with 3,3′-diaminobenzidine (DAB, Sigma-Aldrich), and the footpad sections were mounted on gelatin-coated slides for additional analyses.

### Quantification of immunohistochemical profiles

#### Quantification of different phenotypic DRG neurons

Each DRG section was systematically photographed at 200× magnification (Axiophot Microscope, Zeiss) to produce a montage of the entire DRG section in accordance with an established procedure (Hsieh et al., 2012a; Hsieh et al., 2008; Lin et al., 2013). Ganglia neurons were grouped, and they were larger than other cell types such as fibroblasts in a DRG section. To identify the labeled neurons, optical intensities between the immunoreactive and background neurons were determined. On a 0–255 scale, preliminary analysis showed the optical intensities of FITC to be 138–255. Similarly, the optical intensities of TR were 111–248, and the intensities of AMCA were 155–239. Each signal of a fluorochrome below these ranges acted as background. To avoid bias, only neurons with a clear nuclear profile and with an intensity threshold that met the criteria after labeling were counted, and only the areas containing ganglia neurons were measured using ImageJ (version 1.44d, National Institutes of Health).

#### Quantification of CGRP(+) IENFs

CGRP(+) IENFs were counted under 400× magnification (Axiophot Microscope, Zeiss). The counting protocol accorded with previously established criteria in a coded fashion (Hsieh et al., 2000; Hsieh et al., 2008; Hsieh et al., 2012b). The fibers with branching points in the epidermis were counted as a single intraepidermal fiber; fibers with branching points in the dermis were each counted as single intraepidermal fibers. The length along the lower margin of the stratum corneum was defined as the epidermal length and determined (ImageJ version 1.44d). IENF density was defined as the number of counted IENFs divided by the epidermal length (fibers/cm).

### Statistical analysis

To remove individual bias, five to eight animals were used in each group, and the coding information was masked during the behavioral tests and quantification procedures. All data were expressed as the mean±standard deviation of the mean, and an unpaired *t*-test was performed for data with a Gaussian distribution. For data that did not follow a Gaussian distribution, a nonparametric Mann–Whitney test was conducted. In the behavioral studies, two-way repeated measure analyses of variance were performed followed by a Tukey *post hoc* test when a significance level of *P*<0.05 was obtained.

## Supplementary Material

Supplementary information
