# Supplementary material for: Treatment with methyl-β-cyclodextrin prevents mechanical allodynia in resiniferatoxin neuropathy in a mouse model
Source: Biol Open. 2018 Dec 21;8(1):bio039511. doi: 10.1242/bio.039511 (PMC6361210; doi:10.1242/bio.039511)
Supplement: Supplementary information [file biolopen-8-039511-s1.pdf]

Biology Open (2019): doi:10.1242/bio.039511: Supplementary information

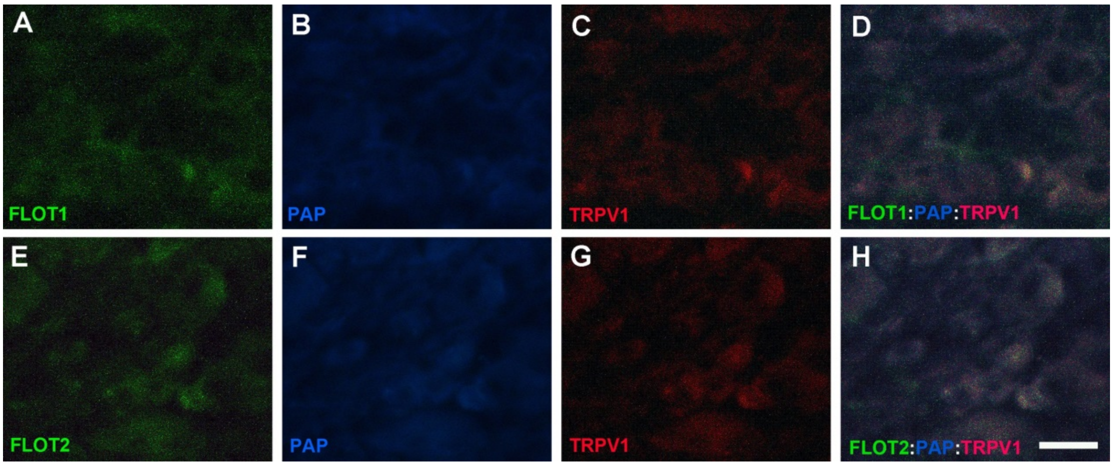

Figure S1. Antibody validation with no primary antibody control in the dorsal root ganglion sections.

Triple-labeling immunofluorescent staining was performed without primary antisera while preserving corresponding conjugated secondary antisera in two combinations: (1) (A–D) anti-flotillin 1 (FLOT1; A and D), anti-prostatic acid phosphatase (PAP; B and D), and anti-transient receptor potential vanilloid subtype 1 (TRPV1; C and D) antisera; (2) (E–H) anti-flotillin 2 (FLOT2; E and H), anti-PAP (F and H), and anti-TRPV1 antisera (G and H). Merged images are in (D) FLOT1:PAP:TRPV1 and (H) FLOT2:PAP:TRPV1.

Bar, 25 µm.

Biology Open • Supplementary information

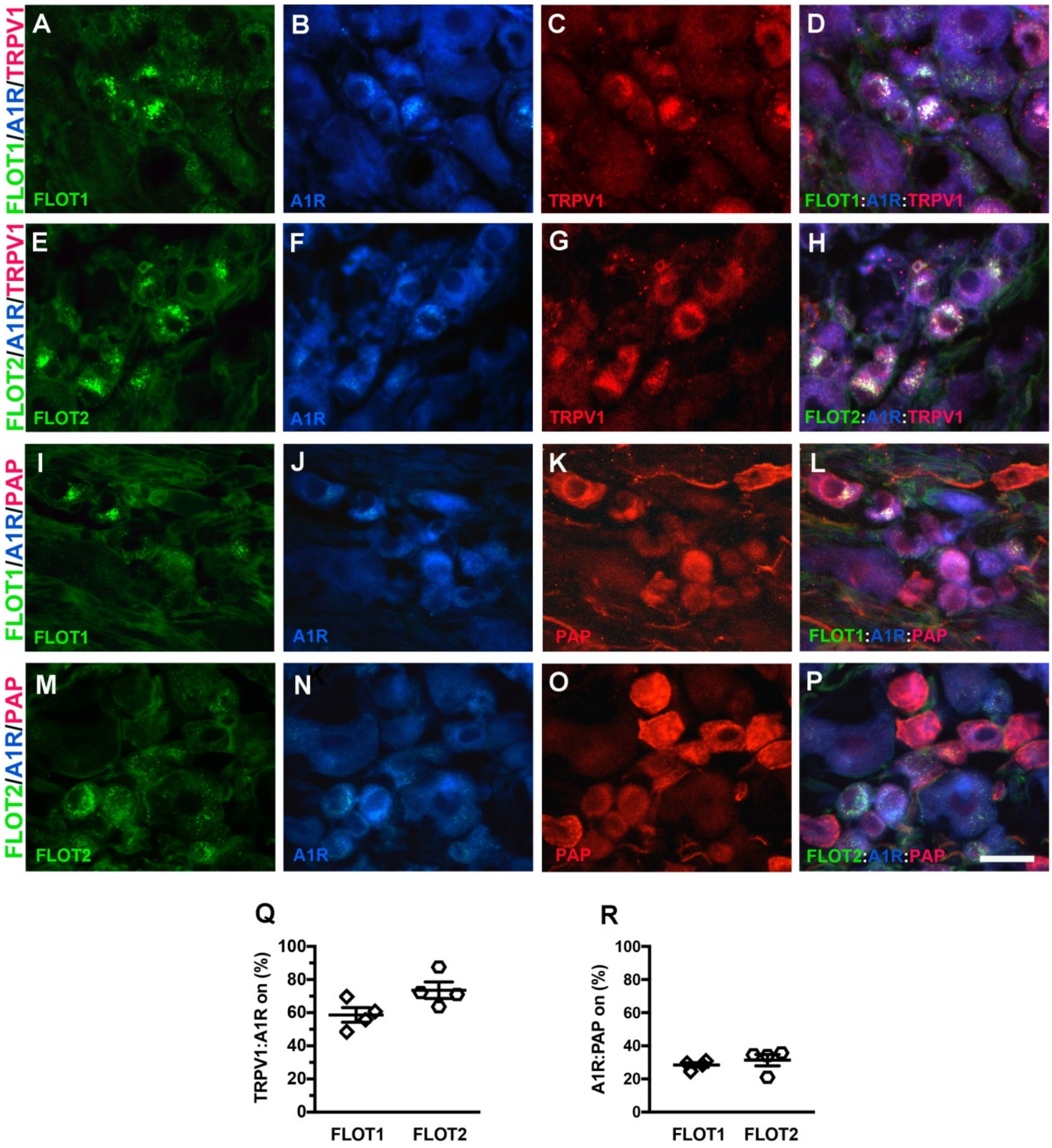

Figure S2. Different phenotypic molecular compositions on a membrane microdomain.

Triple-labeling immunofluorescent staining was used to investigate the molecular compositions on a membrane microdomains. (A–D) Anti-flotillin 1 (FLOT1; A and D), adenosine A1 receptor (A1R; B and D), and transient receptor potential vanilloid subtype 1 (TRPV1; C and D) are colocalized on the dorsal root ganglia neurons. (E–H) Colocalized patterns of flotillin 2 (FLOT2; E and H), A1R (F and H), and TRPV1 (G and H). (I–P) Similar approaches applied to the colocalization of FLOT1, A1R, and prostatic acid phosphatase (PAP) (I–L) and FLOT2, A1R, and PAP (M–P). (Q) Ratio of TRPV1(+):A1R(+) neurons colocalized on FLOT1(+) (opened diamond, n = 4) and FLOT2(+) neurons (opened hexagon, n = 4) according to Fig. S2A–H. (R)

Biology Open • Supplementary information

Ratio of A1R(+):PAP(+) neurons colocalized on FLOT1(+) (opened square, n = 4) and FLOT2(+) neurons (opened hexagon, n = 4) according to Fig. S2I–P. TRPV1 and A1R colocalized with FLOT1 and FLOT2 to a higher level than A1R and PAP. Bar, 25 µm.

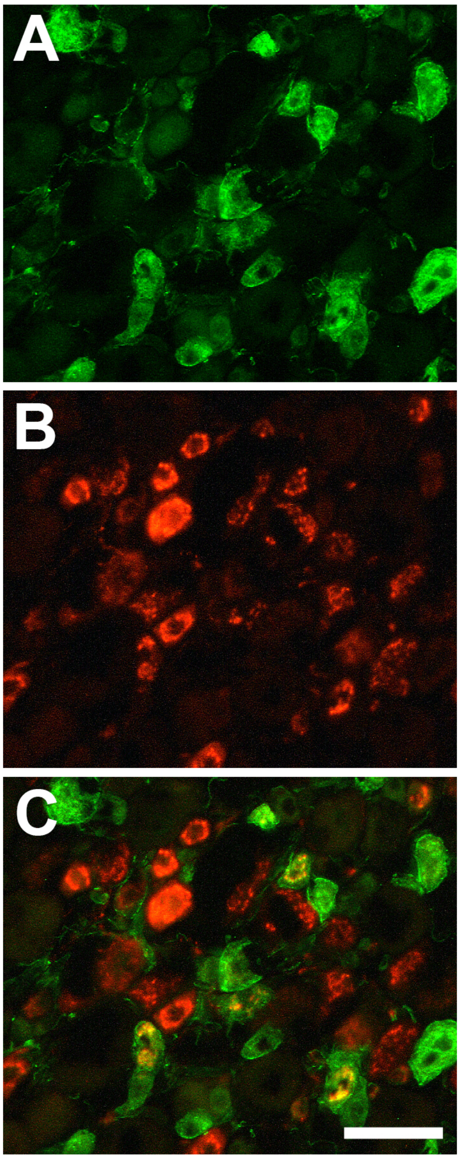

Figure S3. Colocalized profiles of prostatic acid phosphatase (PAP) and calcitonin gene-related peptide (CGRP) in the dorsal root ganglion (DRG) neurons.

(A–C) Double-labeling immunofluorescent staining performed using anti-PAP (A and C in green) and anti-CGRP antisera (B and C in red) in the DRG of the vehicle group.

(C) PAP and CGRP merged for analysis.

Bar, 50 µm.

Biology Open • Supplementary information
